# The lipid droplet—a well-connected organelle

**DOI:** 10.3389/fcell.2015.00049

**Published:** 2015-08-12

**Authors:** Qiang Gao, Joel M. Goodman

**Affiliations:** Department of Pharmacology, University of Texas Southwestern Medical CenterDallas, TX, USA

**Keywords:** lipid droplet, organelle junction, protein trafficking, endoplasmic reticulum, mitochondria

## Abstract

Our knowledge of inter-organellar communication has grown exponentially in recent years. This review focuses on the interactions that cytoplasmic lipid droplets have with other organelles. Twenty-five years ago droplets were considered simply particles of coalesced fat. Ten years ago there were hints from proteomics studies that droplets might interact with other structures to share lipids and proteins. Now it is clear that the droplets interact with many if not most cellular structures to maintain cellular homeostasis and to buffer against insults such as starvation. The evidence for this statement, as well as probes to understand the nature and results of droplet interactions, are presented.

## Introduction

Cytoplasmic lipid droplets (usually shortened to “droplets” hereafter) are virtually ubiquitous in eukaryotic cells and exist even in prokaryotes (Alvarez and Steinbüchel, 2002; Chapman et al., 2012; Walther and Farese, 2012). They dominate the cytoplasm of certain normal cells, such as those of plant oil seeds, fungal cells growing on lipid sources, and adipocytes and cells of the fat body in animals. Lipid droplet-packed cells are the hallmarks of two common human diseases: foam cells in atherosclerotic plaques, and hepatic parenchymal cells in fatty liver (Yuan et al., 2012; Sahini and Borlak, 2014). Although in the light microscope one observes apparently free-standing coalescent spherical units of translucent material that stain with lipid dyes such as Oil Red O, early ultrastructural studies revealed a thin phospholipid membrane encircling the lipid core that further analyses indicated was a single phospholipid leaflet (Tauchi-Sato et al., 2002). Moreover, subjecting the “fat cake” formed from centrifuging adipose tissue homogenates to SDS gels revealed a protein component of droplets (Greenberg et al., 1991). Early proteomic studies of isolated droplets (Athenstaedt et al., 1999; Brasaemle et al., 2004) confirmed a rich assortment of droplet-associated proteins, many of which were already known to play roles in generation and breakdown of neutral lipids (reviewed in Yang et al., 2012).

These proteomic studies also revealed highly specific markers of other organelles, such as ER luminal chaperones and components of mitochondrial oxidative phosphorylation. That ER and mitochondrial proteins were so frequently associated with lipid droplets, which are typically purified through several rounds of flotation in aqueous buffers under conditions in which other organelles pellet, suggested that they represented more than contaminants adventitiously adhering to droplets during fractionation.

**Figure 1:**
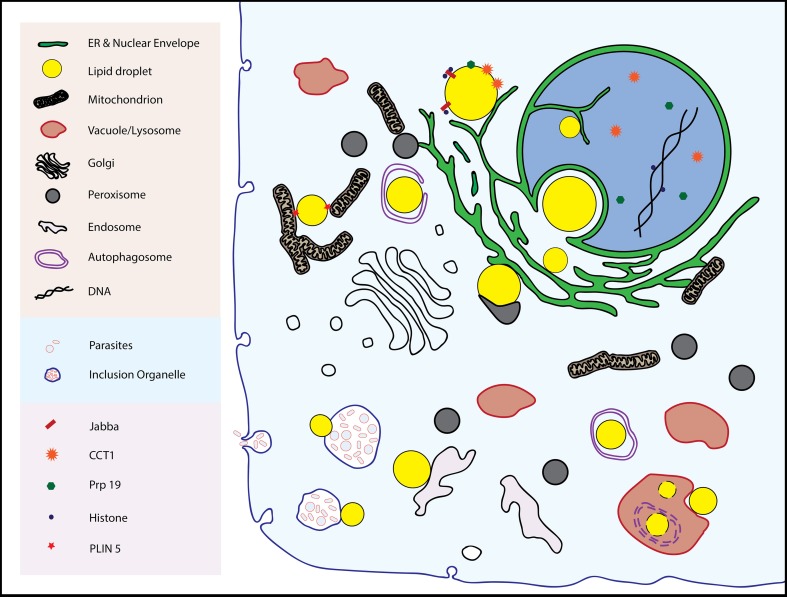
**The multitude of inter-organellar interactions involving lipid droplets are shown**. See text for details.

Inter-organellar junctions are proving to be the rule, not the exception, in cell biology. Examples of stable or dynamic associations include junctions between the ER and several organelles including mitochondria, plasma membranes, vacuoles/lysosomes, Golgi, and endosomes (reviewed in Helle et al., 2013) Peroxisomes, components of which form at the ER, have contacts with lysosomes, which may be of fundamental importance in cholesterol transport (Chu et al., 2015). Lipid droplets also form associations with these organelles (with the possible exceptions of Golgi and plasma membrane), the subject of this review (Figure 1). Whether these physical connections between organelles have physiological importance is now a tractable question as proteins specific to junctions are being identified, and reverse genetics used to probe their function by observing phenotypes in their absence.

Droplets form stable associations of demonstrated physiological value at least with the ER and mitochondria, and the nature of these connections are a large part of this review. However, droplets also appear to bind to other organelles such as the inner nuclear envelope, lysosomes/vacuoles, and endosomes. Evidence for these connections are also presented here with speculation (from us and others) about their relevance.

## Relationship with the ER

### Introduction

Cytoplasmic lipid droplets, as well as secreted lipoproteins, originate in the ER. But the relationship does not end there. Associations between droplets and the ER, first observed by electron microscopy 35 years ago (Novikoff et al., 1980), remain. In yeast, droplets do not appear to ever dissociate from the ER (Szymanski et al., 2007), although in mammals there may be two distinct populations, one attached to the ER, the other not (Wilfling et al., 2013). Membrane bridges between ER and droplets were also observed in this work, although the molecular composition of the bridge remains to be determined. Formation of such contact zones is hypothesized to involve the Arf1-COPI complex (see below; Table 1 lists proteins that play important roles in the interactions of droplets with organelles).

**Table 1:** **Proteins implicated in lipid droplet interactions with other organelles**.

**Organelle**	**Protein**	**Description**	**References**
Endoplasmic reticulum	Arf-COPI components	Its action may result in ER tethering	Wilfling et al., 2013
	Seipin	Important for droplet biogenesis	Cartwright et al., 2015
	FIT2	Important for TAG transfer to droplets	Miranda et al., 2014
	Lipin	May provide DAG for droplet assembly	Sim et al., 2012
	Lro1p	Produces TAG at the ER/droplet interface	Wang and Lee, 2012
	UBXD2, UBXD8/Ubx2p, p97/VCP, AUP1,	ERAD proteins often found on droplets, connecting function of the two organelles	Suzuki et al., 2012; Olzmann et al., 2013
Mitochondria	PLIN5	Mediates droplet–mitochondrial interactions, modulates droplet lipases	Mason and Watt, 2015
Peroxisomes	SDP1	Lipase in plants that traffics between peroxisomes and droplets	Thazar-Poulot et al., 2015
Nucleus	Histones, Jabba	Certain histones stored on droplets	Welte, 2015; Wang et al., 2012
	CIDE family proteins CCT1	Control transcription when not bound to ER or droplets Shuttles from nucleus to droplets to affect phospholipid synthesis	Guo et al., 2008; Krahmer et al., 2011
	Prp19	Found on LDs. In nucleus controls many processes	Cho et al., 2007
Lysosomes/yeast vacuoles	Core autophagy machinery	Mediates lipophagy	van Zutphen et al., 2014
Endosomes	RAB5	Mediates binding of droplets to endosomes *in vitro*	Liu et al., 2007
Parasitic vacuoles	No known factors		
Droplet (homotypic)	Fsp27	Mediates droplet fusion	Gong et al., 2011
	RAB8A	Mediates Fsp27 function	Wu et al., 2014

Besides its involvement in droplet assembly, the functions of ER–droplet connections that explain their stable nature likely include protein and lipid trafficking, response to ER stress, and a role in ER-associated degradation (ERAD).

### Droplet assembly

There are several recent reviews on lipid droplet formation (Gross and Silver, 2014; Pol et al., 2014; Wilfling et al., 2014a; Hashemi and Goodman, 2015). Neutral lipids are initially generated by enzymes in the ER. As droplets form, some of these proteins such as isozymes of glycerol-3-phosphate acyltransferase (GPAT) and diacylglycerol acyltransferase (DGAT) partially or fully transfer to the droplet surface (Jacquier et al., 2011; Wilfling et al., 2013). Time-lapse images showed droplets emanating from the perinuclear ER ring in yeast, a process catalyzed by seipin (Cartwright et al., 2015). How seipin initiates droplet formation is still obscure, although it may serve as a scaffold for enzymes in the pathway of neutral lipid synthesis, such as the phosphatidate hydrolase lipin (Sim et al., 2012). FIT2, an ER protein that binds triacylglycerols, likely also contributes to droplet formation (Gross et al., 2011; Miranda et al., 2014). Cytoplasmic proteins such as PLIN3 (perilipin 3/Tip47), may facilitate membrane curvature that must accompany droplet formation (Skinner et al., 2009). How these factors, and those still-to-be identified, coordinate their function, is still unknown, although seipin may act as a binding scaffold (Talukder et al., 2015).

Droplets do not always form *de novo*. Pre-lipid droplets on the ER exist on starved mammalian cells that are the loci for new droplet assembly when incubated with fatty acids (Kassan et al., 2013). Droplets may also form by fission, as documented in *Schizosaccharomcyes pombe* (Long et al., 2012). Care was taken to rule out z-section artifact in this study, which showed a small droplet emanating from a larger one. Interestingly, the young droplet was not adjacent to the ER during this process suggesting that it may begin its life independent of the ER. Small droplets also appear to form from a large one during acute lipolysis (Marcinkiewicz et al., 2006), although these new organelles are likely a product of *de novo* synthesis rather than fragmentation, based on evidence from time-lapse microscopy (Paar et al., 2012).

### ER to droplet trafficking

A subset of proteins traffic to droplets via the ER, as covered in a recent review (Walther and Farese, 2012). Although we are not aware of any study showing trafficking of endogenous proteins in cells at steady state (i.e., without induction of droplet synthesis or overexpression of cargo), the evidence is strong that this pathway exists. Several proteins such as caveolins have distinct ER and droplet targeting domains (Ingelmo-Torres et al., 2009); When separate, deletion of the droplet-targeting domain results in ER localization. Deletion of the ER localization domain results either in failure to target to either organelle, or targeting to droplets via the cytosol. Ancient Ubiquitous Protein 1 (AUP1) is an exception with overlapping ER and droplet targeting domains (Stevanovic and Thiele, 2013). Another line of evidence is in systems in which droplet formation is stimulated, by incubation of cells in oleic acid or induction of a neutral lipid-synthesizing enzyme. Before stimulation, several droplet proteins have been shown to accumulate in the ER. They then migrate to droplets upon induction (Jacquier et al., 2011; Thiel et al., 2013; Wilfling et al., 2013). Two motives for droplet targeting have been established: amphipathic helices and hydrophobic hairpins (Thiam et al., 2013b), although other motifs can target as well (Murugesan et al., 2013). Some of these may simply bind other resident droplet proteins. It has been difficult to derive the rules for a prototypic “lipid droplet targeting motif.” There is even less known about how this signal (singular or plural) is/are specifically recognized at the droplet, and whether this recognition has its origin in a classical receptor/docking complex or is a product of the physicochemical nature of the droplet phospholipid monolayer and underlying neutral lipid core. Whatever the rules, this process is conserved: mammalian and plant droplet proteins can target efficiently in yeast and even induce droplet formation (Jacquier et al., 2013). There is evidence supporting the role of phospholipid density and surface tension on droplets controlling trafficking of proteins (Thiam et al., 2013b). Related questions remain: How do droplet proteins initially enter the ER? Thus far there are no examples to our knowledge of endogenous droplet proteins containing traditional signal peptides that are cleaved during initial translocation across the ER membrane. It is reasonable that droplet proteins that originate in the ER bypass the SEC61/signal peptidase system, which is designed for secreted or transmembrane (with hydrophilic domains on both sides) proteins, both incompatible with the droplet topology. Is there a unique targeting pathway into the ER for droplet proteins? Trafficking from ER to droplets occurs over several minutes (Jacquier et al., 2011), suggesting that there may be more involved than simple lateral diffusion. What is the rate-limiting step in trafficking? No doubt studies in the near future will address these issues.

A layer of regulation in ER–droplet protein trafficking has been discovered in yeast in which the growth phase affects the partitioning of the diacylglycerol (DAG) acyltransferase, Dga1p, between these two organelles. Targeting of Dga1p from ER to droplets was first described in a system in which droplet assembly was induced (Jacquier et al., 2011). In more recent work, the regulation was uncovered: In early log phase, when DAG is largely channeled into phospholipids, Dga1p is relatively inactive in the ER. As cells approach stationary phase, it is transported to lipid droplets for triacylglycerol synthesis. Return of Dga1p to the ER is promoted by Ice2p (previously known to be involved in the inheritance of ER), which also coordinates the use of DAG for phospholipid synthesis (Markgraf et al., 2014).

Besides the trafficking of endogenous proteins, trafficking of viral proteins from ER to droplets is required for the assembly of *Flaviviridae* family viruses, notably hepatitis C (HCV) and Dengue viruses, as well as for other viral families (Saka and Valdivia, 2012). Droplets promote the assembly of viral capsids by attracting amphipathic helices of the viral proteins to their surfaces. This has been best studied in HCV, in which the core protein first enters the ER via a cleaved signal sequence (presumably through SEC61!), before migrating to droplets, where it then attracts other viral proteins. One of these is NS4B, which contains both ER and lipid droplet targeting signals. Surprisingly, in the absence of the ER hydrophobic signal, the protein appears to go directly to droplets from the cytosol, as determined by fluorescence microscopy (Tanaka et al., 2013).

It should be noted that there are routes to droplets other than through the ER. Evidence supports a direct path from cytosol to droplets for CCT1 (phosphocholine cytidylyltransferase) which likely traffics there depending on the phospholipid density on the droplet surface (Krahmer et al., 2011) and the exchangeable perilipins (perlipins are a group of droplet-associated proteins that share a common domain) PLIN3, PLIN4, and PLIN5 (Wolins et al., 2006). The trafficking of the adipose triglyceride lipase, ATGL, to droplets depends on the COPI pathway of retrograde protein transport (Beller et al., 2008), but the mechanistic relationship (whether it is direct or indirect) is not clear. RAB18 presumably binds to droplets from the cytosol through its isoprenoid modification, similar to other Rab proteins. Finally, a triacylglycerol (TAG) lipase in *Arabidopsis thaliana*, SDP1, can transit to droplets from peroxisomes via a retromer complex (previously known to transport proteins from endosomes to the trans-Golgi network) during seed development (Thazar-Poulot et al., 2015).

Bridges between the ER and droplets should allow transfer of phospholipid and neutral lipids between these two compartments. Experiments are lacking to probe for barriers to lipid trafficking at ER/droplet junctions or the extent to which new lipid synthesis is concentrated at junctions. The yeast diacylglycerol acyltransferase Lro1p is localized to ER/droplet junctions, suggesting that synthesis from this source is indeed coupled to droplet expansion (Wang and Lee, 2012).

### ER stress and droplets

There is a conserved correlation between ER stress and an increase in lipid droplets. In yeast, the knockout of genes involved in the protein glycosylation pathway or by administration of tunicamycin or brefeldin A (strong inducers of ER stress), resulted in an increase in the number of lipid droplets and often an increase in neutral lipids (Fei et al., 2009). These effects are not caused by the classical stress response pathway because they occur even in the absence of the conserved UPR initiator Ire1p. Most likely there is a rerouting of precursors such as phosphatidic acid and diacylglycerol from phospholipid to neutral lipid synthesis. Consistent with this, the anterograde inhibitor, brefeldin A, was found to cause an increase in lipid droplets at the expense of phospholipid synthesis (Gaspar et al., 2008). Brefeldin A treatment also resulted in an increase in TAG and lipid droplets in *Clamydomonas* and the related alga, *Chlorella vulgaris* (Kim et al., 2013).

In mammals, ER stress is linked to liver steatosis. Mice knocked out in a key ER stress component, ATF6α, were more prone to accumulation of liver lipid droplets, found to be caused by a combination of lower β-oxidation, less lipoprotein secretion, and an upregulation of adipogenic genes (Yamamoto et al., 2010).

The synthesis of droplets may be a protective mechanism to prevent aggregation of misfolded proteins as a result of ER stress (Welte, 2007). The fundamental question in all these systems is the mechanism by which lipids are re-routed from membrane synthesis to storage of neutral lipids, a question addressed by studies of ER-assisted degradation.

### ER-assisted degradation (ERAD)

ERAD is elicited by an accumulation of unfolded or misfolded proteins in the ER. There is growing evidence that ERAD is intimately tied to lipid droplets and control of neutral lipid accumulation.

Several proteins that function in ERAD to extract proteins from the ER are colocalized to ER and droplets. These include derlin-1, UBXD2, UBXD8, p97/VCP, and AUP1 (Suzuki et al., 2012; Olzmann et al., 2013; Stevanovic and Thiele, 2013). Evidence for colocalization is their appearance in proteomes of isolated droplets, and live and fixed cell fluorescence with antibodies or tagged proteins. Proteome evidence requires caution since classical luminal ER markers (such as Hsp70/BiP) often copurify with droplets and probably represent tightly-bound ER fragments. Yet fluorescence microscopy is compelling that ERAD proteins can localize around droplets. Ultrastructural studies usually do not accompany most of these reports, but it seems likely, based on a report with apolipoprotein B (see below) and the transmembrane topology of ERAD components that they are localized to a specialized region of the ER that surrounds droplets.

The trafficking of UBXD8 between bulk ER and droplets has been studied in detail (Olzmann et al., 2013). The ER protein UBAC2 retains UBXD8 in that compartment normally, but releases it to translocate to droplets upon the addition of oleate to the medium. Addition of oleate also causes trafficking of p97/VCP to the droplet, which depends on its direct binding to UBXD8.

Proteins destined for degradation by ERAD colocalize with droplets, notably HMG-CoA reductase and poorly lipidated apolipoprotein B-100 (Ohsaki et al., 2006; Hartman et al., 2010). For reductase, a small fraction of protein destined for degradation in the presence of cholesterol copurifies with isolated droplets (Hartman et al., 2010), although it is not clear if this fraction is a kinetic intermediate directly en route to degradation from the bulk ER. Apolipoprotein B accumulates around droplets if proteosomal degradation is blocked (Ohsaki et al., 2006), suggesting that this is the normal site for degradation. Ultrastructural studies indicate that the apolipoprotein accumulated with the block is contained within ER membranes and other structures that are tightly associated, but distinct from the droplet phospholipid monolayer (Suzuki et al., 2012).

To probe whether droplets are functionally important for ERAD, neutral lipid synthesis was inhibited by triacin C, a blocker of acyl-CoA synthetases. The number of droplets was reduced by 40% and was accompanied by a slower rate of degradation of three ERAD substrates, suggesting that droplets are important for ERAD. Surprisingly, knockdown of AUP1, a member of the ERAD complex, resulted in fewer lipid droplets, linking ERAD to droplet formation (Klemm et al., 2011). Conversely, trafficking of UBXD8 to droplets resulted in an increase in neutral lipid due to inhibition of the triglyceride lipase, ATGL, by promoting dissociation of its activator CGI-58 (Olzmann et al., 2013). Similarly, deletion of *UBX2* (yeast UBXD8) resulted in reduced levels of triacylglycerol (Wang and Lee, 2012).

These experiments show that ERAD and droplet lipid metabolism are intimately related. An early model hypothesized that droplets could be an escape hatch used by the ER through which unfolded proteins gain access to ERAD and degradation (Ploegh, 2007). However, in yeast, knocking out neutral lipid biosynthetic enzymes, and thereby eliminating visible droplets, had no effect on ERAD (Olzmann and Kopito, 2011). Although one can interpret these results as indicating that yeast and mammals have fundamentally different mechanisms for ERAD, it is more plausible that both systems share common droplet elements (for example, droplet-associated proteins that do not require droplets *per se*) that have not yet been elucidated.

## Role of Arf1-COPI

A discussion of the Arf1-COPI complex is relevant since it can catalyze intra-organellar communication. The retrograde transport of cargo between Golgi stacks and from the cis-Golgi to the ER is mediated by Arf1-COPI machinery; details have been well worked out (Beck et al., 2009). An early report identified Arf1 (which is an adaptor for COPI coat binding to nascent vesicles) as a binding protein to PLIN2 and showed that a dominant negative mutant of Arf1, or the COPI poison brefeldin A, led to dissociation of PLIN2 from droplets (Nakamura et al., 2004).

More recently, components of COP1-mediated retrograde transport were found in an RNAi screen in *Drosophila* S2 cells for factors involved in droplet assembly or morphology. These included the Arf1 homolog, *Arf79f*, the Arf GEF (GTP exchange factor), *garz*, and several coat components. Cells from these knockdown strains contained larger and more dispersed droplets reflecting an increase of neutral lipid in these cells. In contrast, no gain-of-lipid phenotype was seen for RNAi knockdowns of COPII or clathrin coat subunits, suggesting an involvement of Arf1-COPI in the regulation of lipid droplet morphology and metabolism (Guo et al., 2008). The authors suggested possible functions in droplet budding (analogous to vesicle budding) or lipolysis. In an independent study, COPI was shown to be important for controlling neutral lipid levels in both mammalian and fly cell cultures (Beller et al., 2008). Knockdown of expression of COPI or Arf1 subunits, or administration of brefeldin A resulted in a large decrease in the droplet-associated lipase ATGL in this study. The effect is likely the cause of the larger droplets in COPI-knockdown cells since there was no further increase in neutral lipids if ATGL were knocked down in these cells. In addition, the authors found inappropriate colocalization of PLIN2 and PLIN3 on droplets. Normally, PLIN3 localizes only to smaller droplets while PLIN2 associates with larger ones. In a more recent study, GPAT4 was found to poorly localize to droplets in COPI-knockdown cells (Wilfling et al., 2014b).

A mechanistic explanation for the protein targeting defects in COPI-deficient cells was recently proposed (Thiam et al., 2013a). The authors developed an elegant inverted lipid droplet system in which phospholipid-lined aqueous droplets float in a sea of neutral lipids. The addition of GTP and Arf1-COPI components resulted in the budding of 60-nm nanodroplets into the aqueous phase, which led to an increase in the monolayer phospholipid surface tension, promoting fusion with other inverted droplets (Thiam et al., 2013a). The idea was further developed in intact *Drosophila* S2 cells (Wilfling et al., 2014b). In this study, depleting Arf1-COPI resulted in an increase in levels of phosphatidyl choline (PC) and phosphatidyl ethanolamine on the droplet surface, and the decrease in surface tension caused a delay in the recruitment of the CTP:phosphocholine cytidylyltransferases, CCT1 and CCT2, to LDs. Furthermore, the work suggested that by raising surface tension by removing phospholipids, the Arf1-COPI machinery controlled not only protein targeting to droplets but the development of LD/ER bridges through which GPAT4 traffics (Wilfling et al., 2014b).

## Droplets and mitochondria

### Direct and indirect communication

Close associations of lipid droplets with mitochondria are well known and seen in a variety of cell types including adipocytes, lactating cells, myotubes, and oocytes (summarized in Goodman, 2008). Junctions between these two organelles expand with an increased need for energy, for example, in exercising muscle (Tarnopolsky et al., 2007). It is logical to conclude that droplet/mitochondrial synapses allow the direct flow of fatty acids from neutral lipid stores to the mitochondrial matrix for β-oxidation to meet the cell's energy needs. However, since enzymes for reacylation of fatty acids are found in mitochondria (see Bosma et al., 2012), there may be two-way trafficking of lipids.

### PLIN5 and fatty acid flux

A key player in establishing the droplet mitochondrial junction is PLIN5. Expression of this protein drives mitochondria to lipid droplets (Wang et al., 2011). Mitochondrial binding depends on the C-terminus of the perilipin; ablation of the last 20 amino acids is sufficient to prevent mitochondrial aggregation onto droplets. The binding partner on the mitochondrial surface has not yet been determined.

There has been intense interest in the past few years regarding the role of PLIN5 in lipolysis of triacylglycerol from droplets (reviewed in Mason and Watt, 2015). The consensus is that PLIN5 normally serves as a barrier to lipolysis. PLIN5-knockout animals rapidly lose neutral lipid upon fasting, and PLIN5 overexpression results in larger triacylglycerol stores. However, PLIN5 is responsive to PKA stimulation: Upon PKA activation, lipolysis increases in heart tissue, and this is blocked by mutation of the consensus PKA phosphorylation site of the perilipin. PLIN5 can bind to both HSL (hormone-senstivie lipase) and ATGL as well as the ATGL activator, CGI-58, and phosphorylation likely displaces CGI-58 from PLIN5, allowing it to activate ATGL (Pollak et al., 2015).

Interestingly, overexpression of PLIN5 in skeletal muscle results in the protein localizing not only to droplets but also to the mitochondrial matrix (Bosma et al., 2012). Its function at that location is not known, and, as the protein does not have an obvious mitochondrial targeting signal, its import mechanism is unknown. More studies are needed to determine the physiological significance of its intra-mitochondrial localization.

The droplet–mitochondrial connection may be particularly important during starvation. The balance between lipophagy and cytoplasmic/droplet lipases in providing energy during starvation was probed in mouse embryonic fibroblasts (Rambold et al., 2015). The authors tracked the movement of the fluorescent fatty acid Red-C12 from lipid droplet to mitochondria (for oxidation) after incubating cells in Hank's Balanced Salt Solution without serum or energy source. In these conditions, Red-C12 appeared to move directly from droplet to mitochondria without significant involvement of the lysosome. Interestingly, attached mitochondria had fused, as if to promote fatty acid transfer into a large mitochondrial matrix space. Blocking mitochondrial fusion resulted in less efficient lipid-linked mitochondrial respiration.

## Droplets and peroxisomes

A close association of peroxisomes and lipid droplets was first noted in rabbit ovarian tissue nearly 50 years ago (Blanchette, 1966). Constellations of droplets with surrounding ER, mitochondria and microperoxisomes observed in differentiating 3T3-L1 cells led to the hypothesis that these organelles collaborate in lipid metabolism (Novikoff et al., 1980), an idea that was based in part on the recently discovered ability of mammalian peroxisomes to perform fatty acid β-oxidation [Lazarow, 1978; Lazarow, It had been known considerably earlier that plant glyoxysomes (specialized peroxisomes) could β-oxidize fatty acids (Cooper and Beevers, 1969)]. Studies on peroxisomal/droplet associations were extended to rat fat pads; while peroxisomes were observed close to droplets, direct contacts between the two organelles were not seen (Blanchette-Mackie et al., 1995). A more recent study using COS7 cells revealed a tubular-reticular cluster of peroxisomes, most of which were connected to droplets, especially at the tips of individual peroxisomes in the cluster (Schrader, 2001).

As noted above, the ability of plant peroxisomes to metabolize fatty acids has been known for many decades (Cooper and Beevers, 1969). Glyoxysomes, which metabolize fatty acids to succinate, and which will later transform to leaf peroxisomes, are abundant in oil seeds along with lipid droplets. In a fatty acid 3-ketothiolase mutant (i.e., deficient in fatty acid β-oxidation) in *Arabidopsis thaliana*, large electron-lucent structures are seen within glyoxysomes of etiolated cotyledons that appear to be invaginations from adjacent droplets, and these inclusions contain vesicles (Hayashi et al., 2001). The implication is that these inclusions represent TAG or fatty acids from the droplet that accumulates when β-oxidation is blocked; the structures may be too transient to easily see in wild-type plants.

In fact, neutral lipids as well as phospholipids can be transferred *in vitro* between isolated lipid droplets and peroxisomes, both organelles from cotton (Chapman and Trelease, 1991). The reaction, which involved incubation with radiolabeled lipid, required droplet membrane protein. Transfer of lipid from droplet to peroxisome was confirmed in intact cells by pulse-chase. The authors proposed that normal transfer of lipid from ER to growing peroxisomes involved a droplet intermediate. Droplet protein was necessary for this reaction, as reconstituted droplets devoid of protein were not active in this reaction.

In a recent follow up, a triglyceride lipase, SDP1, in *Arabidopsis* was shown to migrate from peroxisomes to lipid droplets during plant development. Trafficking depended on the retromer complex; mutants of retromer subunits resulted in altered droplet morphology (Thazar-Poulot et al., 2015).

In yeast growing on oleic acid, both peroxisomes and lipid droplets enlarge (Veenhuis et al., 1987). In this medium droplets and peroxisomes make extensive contacts with each other. Peroxisomes are observed that wrap around droplets and even insert processes (pexopodia) within them that are enriched in β-oxidation enzymes (Binns et al., 2006). The association of the two organelles is stable at least over several minutes, while it is much more transient in cells that do not rely on fatty acids for growth.

While there are clearly abundant examples of droplet–peroxisomal interactions, and some evidence for transfer of lipid from droplet to peroxisomes, the molecular and topological details remain murky. The lipase that releases fatty acids at the droplet/peroxisomal junction, let alone its regulation, is not known, nor is there any information regarding the mechanism of transfer of fatty acids between these compartments, or the relative significance of droplets compared to other sources of fatty acids for peroxisomal oxidation.

## Associations with the nucleus

Two types of interactions between lipid droplets and nucleus have been described, involving direct physical associations and indirect communication through bioactive molecules. The reader is referred to a recent reference where these interactions are discussed in detail (Welte, 2015).

Lipid droplets have been frequently observed surrounding nuclei in several types of cells and tissues (Blanchette-Mackie et al., 1995; Szymanski et al., 2007). Because droplets emerge from the ER, and the ER is contiguous with the nuclear envelope, such associations are not surprising. However, droplets have recently been observed within the nucleus. Intra-nuclear droplets (nLDs) were first reported by bright field and fluorescence microscopy in rat liver and HepG2 cells using lipophilic dyes (Layerenza et al., 2013). The nLDs were on average smaller than cytoplasmic droplets and apparently distributed randomly in the nuclear matrix. Isolated nLDs contained a higher ratio of free cholesterol and cholesteryl esters to triacylglycerols than cytoplasmic droplets. In an ultrastructural study of human livers taken at autopsy, most nLDs were observed as invaginations of the nuclear envelope, although the rare nLD (seen in about 1% of hepatocytes) was clearly separated from the envelope (Uzbekov and Roingeard, 2013). Nuclear droplets were also observed in yeast although only in cells with mutated or deleted seipin (Cartwright et al., 2015).

The presence, albeit rare, of nLDs should be viewed in the context of intranuclear lipids and lipid metabolism. Intranuclear phosphorylated inositol phospholipids (PIPs) have been known for years and their roles in chromatin remodeling is a subject of active research (Shah et al., 2013). Moreover, many (and maybe most) cell types have a nucleoplasmic reticulum (NR) (Malhas et al., 2011). The NR, derived from the nuclear envelope, may contain a lumen continuous with cytoplasm as well as one with the intermembrane space between the inner and outer nuclear envelope membranes. Thus, nLDs may directly face an intranuclear “cytoplasmic” compartment or actually bud into the nucleoplasm. Whether the nLDs service the nuclear pools of PIPs, or have any important nuclear function, is not known. Interestingly, the frequency of nuclear droplets can be vastly increased in the absence of seipin, suggesting that this protein ensures that newly formed droplets face into the cytoplasm (Cartwright et al., 2015).

Cytoplasmic lipid droplets may physically alter nuclear shape. HepG2 cells cultured with 1 mM fatty acid mixture for 24 h was found to increase the amount of lipid droplets in the perinuclear area, distorting nuclei (Anavi et al., 2015). Lipid peroxidation products, including hydroxyl-alkanals—alkenals, and alkadienals, likely caused by an excess of fatty acids above the amount that could be stored in droplets as well as increased ROS generation from mitochondrial oxidation, caused covalent modification of several nuclear proteins in this study.

Besides nLDs and physical association of cytoplasmic droplets with nuclei, lipid droplets can regulate nuclear events by storing histones, binding to transcription factors, and physically interacting with other proteins that shuttle to the surface of droplets (Welte, 2015). Droplets of *Drosophila* early embryos store certain histones through an interaction with the droplet surface protein Jabba. In the absence of Jabba, the histones are degraded (Cermelli et al., 2006; Li et al., 2012, 2014), suggesting that lipid droplets not only store lipid precursors but also can supply histones for rapid chromatin remodeling.

The CIDE (cell death inducing DFF45-like effector) family proteins, including Cidea, Cideb, and FSP27/Cidec are other examples of nuclear-droplet communication. These proteins colocalize on the ER and droplets (Puri et al., 2007; Liu et al., 2009; Konige et al., 2014). When not bound to droplets, they affect gene expression: Cidea binds to LXR in 3T3-L1 adipocytes (Kulyté et al., 2011), whereas Cidea and FSP27 interact with CCAAT/enhancer-binding protein β (C/EBPβ) in mammary glands and brown adipose tissue (Wang et al., 2012). Moreover, FSP27 on droplets can sequester NFAT5, which otherwise can respond to osmotic stress and regulate osmoprotective and inflammatory responses. The amino-terminal region of NFAT5 can directly interact with FSP27 on droplets, as determined by bimolecular fluorescence complementation, such that the overexpression of FSP27 inhibits the translocation and transcriptional activity of NFAT5 (Ueno et al., 2013). However, overexpression of FSP27 resulted in its accumulation in the cytosol. These data suggest that sequestration of NFAT5 on droplets plays a physiological role in its regulation.

Another protein that shuttles between nucleus and LDs is CCT1 in *Drosophila melanogaster* (Guo et al., 2008; Tilley et al., 2008; Krahmer et al., 2011). CCT1 is the rate-limiting enzyme in the biosynthesis of PC. Two genes encode this enzyme in the fly: CCT1 was originally found to be a nuclear protein, while CCT2 was cytoplasmic (Tilley et al., 2008). Both may play roles in PC biosynthesis or lipid signaling in their “home” compartments. After incubation in medium containing oleate, however, both forms shuttle to the surface of LDs. CCT1 returns to the nucleus upon removal of the fatty acid (Guo et al., 2008; Krahmer et al., 2011). Apparently, when cells are faced with fatty acid overload, they move to LDs to stimulate PC biosynthesis, allow expansion of LDs, and avoid fatty acid-induced lipotoxicity.

Finally, Prp19 colocalizes to the nucleus and lipid droplets. Prp19 (precursor RNA processing 19) is an essential member of the Prp19 complex (PrpC, also known as NTC, or 19 Complex) which plays important roles in mRNA maturation (transcription elongation, splicing and export), genome stability, and protein degradation (Chanarat and SträSSer, 2013). Besides its nuclear localization, it was identified as a component of lipid droplets (Cho et al., 2007). It appears not to shuttle between nucleus and droplets as leptomycin, which blocks nuclear export, did not affect the distribution of PrpC. Knockdown of Prp19 resulted in lower expression of lipogenic enzymes in the 3T3L1 system (Cho et al., 2007). Because PrpC has many nuclear roles, the relationship of Prp19 binding to droplets to its role in lipogenesis has not yet been worked out.

Thus, there are several layers of interactions between nucleus and lipid droplets, and communication can flow in both directions. Future research will likely elucidate the physiological importance of morphological findings (such as nLDs) and work out downstream effects (such as metabolic signaling) of this rich collaboration between two cellular components.

## Droplets and lysosomes/vacuoles

Since autophagy of lipid droplets, lipophagy, was first reported in hepatocytes (Singh et al., 2009a), the interaction between lysosomes (vacuoles in yeast) and lipid droplets became a research highlight in the lipid metabolism area. Lipophagy, as an alternative to lipolysis by droplet or cytosolic lipases, has been reported in yeast, adipocytes, enterocytes, fibroblasts, neurons, and stellate cells (Singh et al., 2009b; Lettieri Barbato et al., 2013; Liu and Czaja, 2013; Khaldoun et al., 2014; van Zutphen et al., 2014; Wang et al., 2014). It can involve autophagosome formation (macrolipophagy), direct interaction of droplet with lysosome (microlipophagy), or chaperone-mediated autophagy (CMA).

Singh et al. found that the autophagy inhibitor 3-methyladenine (3MA), or a mutant *atg5* gene, resulted in an increase in the number and size of lipid droplets, TAG accumulation, and a decrease in fatty acid β-oxidation, suggesting that cells could break down the LDs through autophagy processes. Furthermore, the group showed that the processes needed ATG7-dependent conjugation for recruiting LC-3 to the LDs surface, an initial step of macroautophagy (Singh et al., 2009a). Regulation of lipophagy also requires the coordination of mTORC1 (the mammalian target of rapamycin complex 1) with nutrient-sensitive transcription factors TFEB, p53, and FOXOs, as described in an excellent recent review (Settembre and Ballabio, 2014). The system is controlled by the energy state within the lysosome, which signals across the membrane through the lysosomal acid lipase (LAL, itself controlled by FOXO1) and mTORC1 to transcriptional factors. In addition, dynamin 2 is important for the regeneration of nascent lysososomes by scission of the tubulated autolysosomes in macroautophagy in hepatocytes (Schulze et al., 2013).

To tease out the importance of lipophagy compared with lipolysis outside the lysosome, a study noted above (Rambold et al., 2015) found that cells starved of carbon source derived most of its fatty acids for mitochondrial oxidation from direct transfer from droplets, presumably from a droplet lipase. However, during serum-starvation the group found that lipophagy played a larger role. Precisely how these two pathways are coordinated will be fascinating to uncover.

In contrast to mammalian cells, lipophagy in yeast more closely resembles microautophagy and requires steps to modify the vacuolar membrane for engulfment of LDs (van Zutphen et al., 2014; Wang et al., 2014). All the core autophagy machinery associated genes are necessary for yeast lipophagy except *Shp1, Vps38, Nyv1, Atg11*, and *Atg20*. Tubulin and Vac8, which is involved in multiple vacuolar processes, are also important (van Zutphen et al., 2014). Wang et al. also reported that the existence of a sterol-enriched vacuolar microdomain is important for stationary phase yeast LDs translocation and hypothesized a feed-forward loop to promote stationary phase lipophagy (Wang et al., 2014).

CMA of droplet proteins is an alternative pathway for generating fatty acids. A recent report showed the involvement of PLIN2 and PLIN3 as substrates for CMA, a pathway that was stimulated during starvation (Kaushik and Cuervo, 2015).

Besides serving as a source of energy during starvation via lipophagy, droplets are also important to promote general autophagy of cellular contents. Thus, general autophagy in yeast was severely reduced in the absence of droplets (Li et al., 2015), and the level of autophagosome formation in HeLa cells was decreased in the absence of lipid droplets or the lipase PNPLA5 (Dupont et al., 2014).

## Droplets, endosomes, and Rab proteins

No Rab protein is exclusively localized to droplets, but much of RAB18 is localized there, and its localization is regulated (Martin et al., 2005). Multiple Rab proteins, which catalyze and specify vesicular trafficking, have been identified as members of the lipid body proteome (Yang et al., 2012). Although their function at the droplet remain obscure (other than possibly a site for storage), some progress has been made. An early report demonstrated the ability of Rabs to reversibly traffic to droplets based on their guanine nucleotide-bound state, suggesting that the interaction is physiological (Liu et al., 2007). Moreover, in this report, RAB5 activation caused the binding of isolated droplets to purified early endosomes, and transfected activated RAB5 could also cause binding of these two organelles in cells. This work, although needing more development, suggests that droplet binding to endosomes is physiologically important and is mediated by a Rab protein.

Another Rab protein, RAB8A mediates droplet–droplet associations. Our group observed homotypic associations in yeast growing on oleic acid, in which multiple chains of droplets encircling the nucleus were found, linked to one another by nipple-like connections (Binns et al., 2006); the function of such junctions remains unknown, although they appear stable. Droplets of mammalian cells can contact each other leading to exchange of core lipids from the smaller to the larger droplet in a process that depends on Cidec/Fsp27 (Gong et al., 2011) and is regulated by RAB8A (Wu et al., 2014).

## Droplets and parasitic vacuoles

Finally, there is dynamic interaction between lipid droplets and inclusion organelles (derived from plasma membrane) generated by unicellular parasites. The best studied is the interaction of droplets with the parasitopherous inclusion organelle formed upon entry of *Chlamydia trachomatis* (Cocchiaro et al., 2008). Droplets can be observed entering this structure from the cytosol. Other invaders use lipids derived from cytoplasmic lipid droplets for their nutrition (reviewed in Saka and Valdivia, 2012). Droplets are recruited to vacuoles containing *Mycobacterium leprae* in Schwann cells (Mattos et al., 2011). In addition fatty acids from lipid droplets are incorporated into neutral lipids in tubercle bacilli infecting lung macrophages, promoting the dormant state (Daniel et al., 2011).

## Conclusion

It is apparent that lipid droplets are well connected to many other cellular compartments, and in some cases (notably ER and mitochondrial contacts) molecules have been identified that are important to initiate or maintain the connections. It is assumed that many of these contacts result in transfer of lipids between compartments, and those droplets serve as the source of lipids for membrane expansion, energy production, and signaling. However, the mechanism and extent of activation of lipases by contact sites, and the mode of fatty acid transfer between organelles, remain obscure. The basic mechanisms of droplet initiation and maintenance by the ER are no longer totally obscure but still lack much basic information. The role, if any, for provision of lipids by droplets within the nucleus is a fascinating issue that requires attention, as is the role that droplets perform in exocytic and endocytic trafficking. Finally, the regulation of energy release from droplets during starvation among pathways—general and specific autophagy and *in situ* lipolysis on droplets in the cytosol—is a research area that should provide answers in the near future. The intricate and intimate connections among cellular organelles form the basis of cell function and continue to provide inspiration to those of us working in this area of biology.

### Conflict of interest statement

The authors declare that the research was conducted in the absence of any commercial or financial relationships that could be construed as a potential conflict of interest.
